# Myoclonus improvement after seizures in progressive myoclonic epilepsy type 7: a case report

**DOI:** 10.1186/s12883-024-03625-z

**Published:** 2024-05-23

**Authors:** Pedro Lucas G S B Lima, Paulo R Nobrega, Fernando Freua, Pedro Braga-Neto, Anderson R B Paiva, Thiago Gonçalves Guimarães, Fernando Kok

**Affiliations:** 1 Faculty of Medicine, Federal University of Ceara, Fortaleza, Ceara Brazil; 2 Division of Neurology, Federal University of Ceara, Fortaleza, Ceara Brazil; 3 Centro Universitário Christus, Fortaleza, Ceara Brazil; 4 Neurogenetics Center, Department of Neurology, University of Sao Paulo, Sao Paulo, Brazil; 5 Movement Disorders Center, Department of Neurology, University of Sao Paulo, Av. Dr. Eneas de Carvalho Aguiar, 255, 5th Floor, Room 5084, Cerqueira Cesar, Sao Paulo, Sao Paulo, 05403-900 Brazil

**Keywords:** Myoclonus, Epilepsy, *KCNC1*, Movement disorders, Case report

## Abstract

**Background:**

Progressive Myoclonic Epilepsy (PME) is a group of rare diseases that are difficult to differentiate from one another based on phenotypical characteristics.

**Case report:**

We report a case of PME type 7 due to a pathogenic variant in *KCNC1* with myoclonus improvement after epileptic seizures.

**Discussion:**

Myoclonus improvement after seizures may be a clue to the diagnosis of Progressive Myoclonic Epilepsy type 7.

**Supplementary Information:**

The online version contains supplementary material available at 10.1186/s12883-024-03625-z.

## Background

Progressive Myoclonic Epilepsies (PMEs) are a group of rare and severe conditions characterized by action myoclonus, refractory epilepsy, ataxia and dementia. Most diseases in this group have an autosomal recessive pattern of inheritance [1, 2]. PMEs usually present in late childhood or adolescence. Patients commonly become wheelchair-bound and have a reduced life expectancy.

Progressive Myoclonic Epilepsy type 7, also known as myoclonus epilepsy and ataxia due to potassium channel mutation (MEAK), is associated with the recurrent c.959G > A (p.Arg320His) variant in *KCNC1* and stands out for , relatively easily treatable epilepsy without severe cognitive impairment and autosomal dominant inheritance associated with *de novo* variants [1, 2]. Other variants in EPM7 have been reported to cause epileptic encephalopathy [3].

## Case Report

A 29-year-old woman presented at the age of 10 with upper limb abnormal jerky movements. These movements were triggered by action and worsened in the morning. After two months, she started focal epileptic seizures with left limb tonic postures that progressed to generalized tonic-clonic seizures in most occurrences. Her mean seizure frequency was around 2 to 5 seizures a month and tonic-clonic seizures lasted up to 5 min at most, followed by 30 to 60 min of somnolence.

Interestingly, involuntary movements worsened during the menstrual period and improved after each generalized tonic-clonic seizure (Video). The improvement lasted from 5 to 7 days after each seizure. Improvement in myoclonus was not seen with fever and the patient did not consume alcohol.

Following the myoclonus and seizures onset in early adolescence, progressive impairment in gait and balance was observed. By the age of 21, the patient was wheelchair-bound.

Seizures were controlled with valproate. The patient has been seizure-free since the age of 15. Before presenting at our outpatient clinic, she was on carbamazepine which wasn’t effective. No other anti-seizure drugs had been used before valproate and piracetam (which we discontinued posteriorly due to somnolence).

The patient’s early development was marked by a delay in walking and language (she walked without support at the age of three and spoke more elaborated phrases around four years of age). Her medical history was remarkable only for insulin-dependent diabetes mellitus. Her family history was unremarkable.

**Fig. 1:**
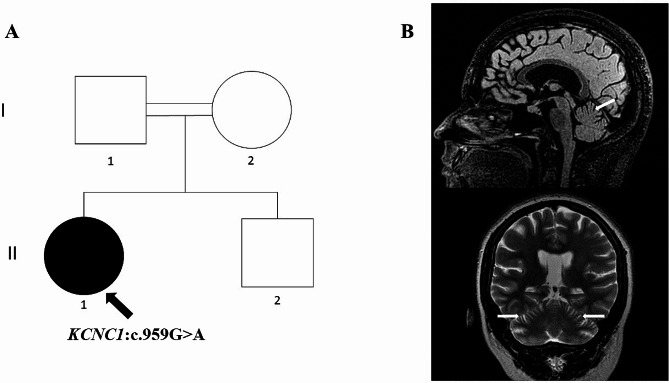
Family pedigree (**A**): the patient’s parents were unaffected first-degree cousins. Brain MRI (**B**) showed mild cerebellar atrophy

By the age of 29, the patient achieved a highschool degree. She recently scored 25/30 on Mini Mental State Examination. Neurological examination revealed severe generalized action myoclonus, global ataxia, and dysarthria (Video). Brain MRI showed mild cerebellar atrophy (Fig. 1). Electroencephalogram disclosed generalized spike and spike-wave paroxysms. Whole exome sequencing identified a previously described missense variant c.959G > A (p.Arg320His) in *KCNC1* [4, 5]. This variant is classified as pathogenic according to ACMG criteria [6] and was reported to cause Myoclonic Epilepsy Type 7 (OMIM#616,187). Unfortunately, parental testing could not be performed for segregation analysis.

## Discussion

Next-Generation Sequencing (NGS) technology has uncovered rare genetic causes in unsolved PME cases, including established and novel PME-related genes, with a diagnostic yield of over 80% in PME, underlining its status as one of the best genetically defined epilepsy syndromes [7].

The potassium channel subfamily Kv3 consists of four subunits which are encoded by *KCNC1, KCNC2, KCNC3*, and *KCNC4*. Pathogenic variants in *KCNC3* are associated with spinocerebellar ataxia type 13, whereas *KCNC2* and *KCNC4* have not been associated with human disease up to this moment [8]. Regarding *KCNC1*, a study conducted in 2016 found that 16 out of 84 clinically confirmed Progressive Myoclonic Epilepsy (PME) cases without clear genetic etiology had a monoallelic missense variant in the *KCNC1* gene, c.959G > A (p.Arg320His), 13 of whom were unrelated [9]. The authors suggested the name MEAK (Myoclonus Epilepsy and Ataxia due to potassium channel mutation) for the condition, corresponding to the PME type 7.

The phenotype described in PME type 7 consists of normal early development with myoclonus as the first symptom (onset at 6 to 14 years), which becomes progressively worse affecting the gait and resulting in patients being wheelchair-bound by adolescence or early adulthood in most cases. Tonic-clonic seizures may be present but are not frequent. Mild cognitive decline occurs in some cases after seizure onset. Magnetic resonance may be normal or show cerebellar atrophy [9, 10]. Exacerbation during menses was reported [5]. High fever is reported to transiently (from hours to days) improve gait and myoclonus in PME type 7 patients [5]. Our patient, however, did not report improvement with fever. Alcohol and pregnancy were also reported to improve the condition of these patients [5]. The patient in this report did not consume alcohol.

Compared to other types of PME, the type 7 has a somewhat similar presentation to Unverricht-Lundborg disease (PME type 1), but with more severe disease progression [2, 10]. To the best of our knowledge, there are no reports describing improvement of myoclonus after seizures in this condition.

Seizures may reduce post-ictal neuronal activity by several mechanisms. Increased calcium entry during depolarization activates a calcium-dependent membrane potassium conductance that allows potassium efflux and membrane hyperpolarization [11, 12]. Extracellular calcium levels also change markedly after paroxysmal neuronal firing. Seizures result in a decline in extracellular calcium activity of approximately 50% [13]. This decrease may impair synaptic transmission because synaptic vesicle fusion and neurotransmitter release are dependent on the entrance of extracellular calcium [14, 15]. Seizure discharges also produce recurrent GABAergic synaptic inhibition via inhibitory interneurons [16, 17], thus reducing excitatory output. Neuromodulators, such as endocannabinoids, adenosine, and neuropeptide Y (NPY) are synthesized and released from neurons following membrane depolarization and inhibit neuronal activity. We hypothesize that the post-seizure inhibition of neuronal activity caused by the aforementioned factors may result in myoclonus improvement in the patient reported here. The deficiency in potassium channel activity may lead to an increase in other compensatory mechanisms responsible for the termination of seizures, but further studies are needed to confirm this hypothesis.

The current therapeutic approach to PMEs remains palliative, to control seizures and myoclonus, and improve patients’ quality of life and independence. Literature is scarce concerning pharmacological options in PMEs, mostly composed of reports and small observational studies [18]. Valproic acid (VPA) is the most reported drug in PMEs. It is described as the drug of choice, alone or in combination with clonazepam, due to its high effectiveness on myoclonus, seizures, and photosensitivity [18].

The PMEs, despite having similar phenotypic features overall, form a very heterogeneous group of conditions, each having a different genetic background and disease-causing mechanism. This makes a single therapeutic approach to all forms unlikely, but also opens up the possibility for personalized therapeutic approaches. Currently, there are several experimental studies in animal and cellular models that aim to correct the main disease-causing mechanism, such as enzyme replacement therapies for Sialidosis and Gaucher’s disease type III, and gene replacement therapy for Lafora disease [2].

This case describes a patient with PME type 7 and illustrates a novel interesting clinical phenomenon myoclonus improvement after seizures in a rare form of progressive myoclonic epilepsy. This adds to the literature as a possible clinical phenomenon that may help clinicians to raise suspicion of Progressive Myoclonic Epilepsy type 7.

### Electronic supplementary material

Below is the link to the electronic supplementary material.

Supplementary Material 1

Supplementary Material 2

## Data Availability

The dataset generated during the current study is available from the corresponding author on reasonable request. The detailed information regarding the identified variant is available in the ClinVar repository under the Variation ID 162519.
