# Supplementary material for: Myoclonus improvement after seizures in progressive myoclonic epilepsy type 7: a case report
Source: BMC Neurol. 2024 May 23;24:169. doi: 10.1186/s12883-024-03625-z (PMC11112770; doi:10.1186/s12883-024-03625-z)
Supplement: Supplementary file 2 — Supplementary Material 2 [file 12883_2024_3625_MOESM2_ESM.pdf]

Video S1. Myoclonus at baseline and improvement after a seizure.
